# Key cellulase components synergizing with lactic acid bacteria to degrade alfalfa lignocellulose to improve lactic acid fermentation

**DOI:** 10.3389/fmicb.2025.1566973

**Published:** 2025-04-15

**Authors:** Linxiong Ma, Junfeng Li, Wanyu Zhao, Jingyu Wang, Yanwen Li, Yueying Xiong, Yongchao He, Xiaohui Chu, Qinhua Liu

**Affiliations:** ^1^Yunnan Provincial Key Laboratory of Animal Nutrition and Feed, Department of Pratacultural Science, Faculty of Animal Science and Technology, Yunnan Agricultural University, Kunming, China; ^2^Institute of Ensiling and Processing of Grass, College of Agro-Grassland Science, Nanjing Agricultural University, Nanjing, China

**Keywords:** lignocellulose, degradation, sugar, lactic acid, key cellulase components

## Abstract

Using cellulase to convert alfalfa lignocellulose into lactic acid (LA) is useful in low-temperature seasons to improve fermentation quality, but it is still unknown which specific cellulase component synergizes with lactic acid bacteria (LAB) to promote LA fermentation. This study aimed to clarify the key cellulase components that synergized with LAB when converting alfalfa lignocellulose into LA during ensiling from late fall to winter (3–20°C) over 140 days. Seven combinations of cellulase component gene-engineered *Lactococcus lactis* (MG1363), cellulase (EN), and a combination of *Lactobacillus plantarum* and cellulase (LPEN) were used as parallel treatments, with a control (CON) without treatment also used. EN degraded lignocellulose best. The pH value in the channel of converting sugars into LA was the key limiting factor for lignocellulose saccharification in LPEN. The optimal combination resulted in the fewest disaccharides (1.02 g/kg DM) and the highest conversion of water-soluble carbohydrates (WSC) to LA, up to 170%. It increased LA content to 80.0 g/kg DM maximally, since cellobiohydrolase better cooperated with MG1363 to ferment lignocellulose into LA than endoglucanase and *β*-glucosidase. Strong LA production was achieved by clarifying key cellulase components with cellulase component gene-engineered LAB.

## 1 Introduction

Grass, primarily consisting of 29–35% cellulose, 26–32% hemicellulose, and 16–21% lignin, is a prevalent renewable biomass (Galkin and Samec, 2016; Ashokkumar et al., 2024). Converting these polymers into chemicals through anaerobic fermentation is a sustainable method to prevent resource competition between the food and chemical sectors (Liu et al., 2020). Lactic acid (LA), a versatile chemical, is widely used in food, leather tanning, cosmetics, and pharmaceutical industries (Martinez et al., 2013; Kim et al., 2022). Approximately 90% of global LA is produced through microbial fermentation (Alexandri et al., 2019; Haag et al., 2016). Due to the increasing demand for LA (12.5%)^1^, and due to the efficiency, environmental friendliness, and high purity obtained from microbial fermentation (Wee et al., 2006), it has maintained its dominant position as the process to achieve LA. Ensiling, an anaerobic fermentation process, relies on naturally occurring lactic acid bacteria (LAB) to convert carbohydrates into LA, lowering pH and preserving the plant material (McDonald, 1981). This process is considered eco-friendly and sustainable for producing LA from lignocellulosic biomass (Bichot et al., 2018; Haag et al., 2016).

Increasing the LA content in silage grass using homofermentative LAB is more effective than hetero-fermentative LAB, because it produces more LA to restrain clostridial fermentation during the ensiling of lignocellulosic biomass. However, this depends on numerous factors, e.g., material and temperature (Kung and Muck, 2015), especially in cold seasons (Cao et al., 2011; Wang et al., 2017). Research on how to use homofermentative LAB to increase LA production and inhibit undesirable fermentation, particularly in high-moisture alfalfa silage, is an interesting research direction.

Most homo-fermentative LAB cannot secrete cellulase to degrade lignocellulose and meet the needs of LA production. Although our group isolated some cellulolytic LAB from the rumen, it did not improve the conversion of WSC into LA (CWL) in ensiled alfalfa due to its poor ability to produce LA (Liu et al., 2020). Li et al. (2018a) discovered that rumen cellulolytic LAB degraded the lignocellulose of Pennisetum sinese and raised LA content. However, when combined with *Lactobacillus plantarum*, they exhibited stronger LA fermentation and cellulase activity. Cellulase consists of endoglucanase (EG), cellobiohydrolase (CBH), and *β*-glucosidase (BG) and hydrolyzes the *β*-1,4-D-glycosidic bonds of cellulose. The complete and successful degradation of cellulose into monosaccharides depends on the composition ratio of cellulase. Applicability of cellulase is influenced by differences in the composition ratio of cellulase (Du et al., 2018). The optimization of the proportions of individual components in cellulase has underscored the significance of lignocellulose hydrolysis. Generally, CBH is sensitive to cellobiose and BG is sensitive to glucose (Decker et al., 2001; Teugjas and Väljamäe, 2013; Sengupta et al., 2024). BG frequently acts as a rate-limiting factor in cellulase degrading cellulose. To eliminate this constraint, the fermentation of sugars by LAB inoculation has been proposed as a method for enhancing lignocellulosic biomass and improving synergy with LAB’s acidolysis. The combination of cellulase composition and LAB was often applied to ensiling grass; however, there were limitations due to their inapposite synergistic effect. Some previous studies have described the negative effects of the combined treatment of cellulase and LAB on alfalfa ensiling, such as ineffectiveness in decreasing DM loss, neutral detergent fiber (NDF), and acid detergent fiber (ADF) and increasing LA content (Kozelov et al., 2008; Lynch et al., 2014). The proposed explanation is that some cellulases cannot degrade alfalfa lignocellulose (Lynch et al., 2014; Li et al., 2022), or some lignocellulose degradation products of cellulase cannot be fermented by LAB (Stokes and Chen, 1994; Tarraran and Mazzoli, 2018). The timeliness of cellulase and LAB was mismatched (Liu et al., 2019). Additionally, there is little information on which cellulase component plays synergistic roles with LAB in fermenting alfalfa lignocellulose into LA.

Our group constructed engineered *L. lactis* subsp. lactis MG1363 (MG1363) strains separately containing the cellulase (*bgl1*, *cbh2,* and *egl3*) gene of *Trichoderma reesei,* and found it could secrete cellulase components (Liu et al., 2019). The combination proportion of engineered MG1363 strains (HT2) containing *bgl1, cbh2,* and *egl3* genes at a ratio of 1:1:1 could secrete cellulase (236 mU/mL FPase activity) (Liu et al., 2019) to degrade the lignocellulose and produce LA (72 g/kg DM) in ensiled alfalfa. Therefore, this study aimed to explore the optimized combination proportion of engineered MG1363 strains to enhance the fermentation quality and to pinpoint key cellulase components that synergistically interact with the host MG1363 during the fermentation of alfalfa lignocellulose, thereby improving LA production. Correspondingly, engineered MG1363 strains containing *bgl*1, *cbh*2, and *egl*3 genes were mixed at 1:1:1, 2:1:1, 1:2:1, 1:1:2, 1:1:0, 1:0:1, and 0:1:1 ratios and were named HT2, HT3, HT4, HT5, E1C1, E1B1, and C1B1, respectively, to use as treatments. Commercial cellulase (EN) and the combination of *Lactobacillus plantarum* and commercial cellulase (LPEN) were used as parallel treatments, with a control (CON) without treatment also used. The lignocellulose degradation, sugar profile, fermentation characteristics, and CWL of high-moisture alfalfa were investigated during ensiling in low-temperature seasons for 140 d.

## 2 Materials and methods

### 2.1 LAB strains, material, and ensiling treatments

The engineered strains of MG1363 containing *bgl1*, *cbh2*, and *egl3* genes of *T. reesei* were constructed by Liu et al. (2019). After culturing in GM17 broth medium at 30°C for 30 h, engineered strains containing *bgl1*, *cbh2*, and *egl3* genes were mixed at ratios of 1:1:1, 2:1:1, 1:2:1, 1:1:2, 1:1:0, 1:0:1, and 0:1:1 and were named HT2, HT3, HT4, HT5, E1C1, E1B1, and C1B1, respectively. The LPEN was a combination of *Lactobacillus plantarum* and commercial cellulase. *Lactobacillus plantarum* was isolated from corn silage, stored at-80°C, and incubated in deMan Rogosa and Sharp (MRS) medium at 37°C for 24 h when used. The EN, the activity of which was 50,000 U/g FPase, was extracted from *T. reesei* and purchased from Ruiyang biotechnology company (Wuxi, China). The HT2, HT3, HT4, HT5, E1C1, E1B1, C1B1, LPEN, and EN were used as the nine treatments.

Alfalfa was planted in three fields (100 m^2^) at Nanjing Agricultural University (Nanjing, China) (Humid subtropical climate, 32°01′ N, 118°50′13.63″ E, 17 masl) on September 25, 2017, and the last harvest time was November 2, 2018. A forage chopper (Sh-2000, manufactured by Shanghai Donxe Industrial Co., Ltd., Shanghai, China) was used to chop the fresh alfalfa into pieces measuring 1 to 2 cm in length. Before ensiling, DM content, pH value, and BC of alfalfa were 279 g/kg FM, 5.15, and 594 mEq/kg DM, respectively. The structural carbohydrate composition of neutral detergent fiber (NDF), acid detergent fiber (ADF), acid detergent lignin (ADL), cellulose, and hemicellulose was 431, 308, 110, 198, and 123 g/kg DM, respectively. The WSC content was 84.3 g/kg DM, and the fraction of individual soluble carbohydrates (g/kg DM) was as follows: disaccharides (24.0 g/kg DM), glucose (10.7 g/kg DM), xylose (13.7 g/kg DM), arabinose (0.21 g/kg DM), and fructose (10.5 g/kg DM). The epiphytic LAB [3.40 lg cfu/g FM] on alfalfa was lower than that of aerobic bacteria (6.97 lg cfu/g FM) and yeasts (5.98 lg cfu/g FM). The fermentation efficiency (FC) of fresh alfalfa was 29.04.

Alfalfa was harvested from three fields (100 m^2^ each). The alfalfa harvested in each field was ensiled with nine treatments (HT2, HT3, HT4, HT5, E1C1, E1B1, C1B1, LPEN, and EN); without treatment was used as the control. Each treatment and the control had four replicates. According to Mcfarland (1907) turbidity standards, inoculation dosage (3 mL aqueous solution) of HT2, HT3, HT4, HT5, E1C1, E1B1, and C1B1 was at 1 × 10^6^ cfu/g FM and were sprayed on alfalfa. The EN is added to take the dose (3 mL aqueous solution) at 2 g/kg of FM. EN and LP were mixed as LPEN, and LPEN was added at a dose (3 mL aqueous solution) of 2 g/kg + 1 × 10^6^ cfu/g FM. Three milliliters of distilled water were added to the control. Alfalfa was mixed with different treatments and then filled into the experimental silo. The experimental silo (polyvinyl chloride bottle, 1 L) was filled with 720 g of treated alfalfa. The silo was sealed with a lid and tape to create an anaerobic condition. All silos were kept at an ambient temperature ranging from 3 to 20°C for 140 days of ensiling, and then the silos were sampled for analysis. Considering this experiment was conducted from late fall to winter using the last alfalfa cut of 2018, the temperature gradually decreased. Consequently, a fermentation period of 140 days was sufficiently long for investigating the enhancing effects of the treatments.

### 2.2 Microbial and chemical analyses

According to the method reported by Liu et al. (2019), the LAB, aerobic bacteria, and clostridia in alfalfa materials were counted after harvesting from the field for 2 h. The BC of alfalfa materials was measured by the method reported by Liu et al. (2018). In brief, 20 g of fresh alfalfa was submerged with 180 mL distilled water for 24 h at 4°C, and the mixture was taken to measure the BC using 0.1 mol/L hydrochloric acid and sodium hydroxide. Hydrochloric acid was added to decrease the pH of the mixture to 3.50 and to eject carbonate. After achieving a pH of 4.00 by adding sodium hydroxide, the quantity of sodium hydroxide required to increase the pH from 4 to 6 was used to calculate the BC (Playne and McDonald, 2010). The DM, NDF, ADF, ADL, and WSC in alfalfa materials were measured by the same method reported by Murphy (1958), Mertens (2002), and AOAC, 2000; Li et al. (2018b). The DM content of fresh alfalfa was determined by oven drying at 70°C for 48 h. The WSC content of fresh alfalfa was assessed using the anthrone method. The NDF content of fresh alfalfa was measured following the AOAC Official First Action method, which includes the use of heat-tolerant alpha-amylase and sodium sulfite. The ADF was measured according to AOAC method 973.18 as outlined in AOAC (2000). Both procedures were adapted for the ANKOM filter bag technique. The procedures were conducted sequentially, and the results are presented on an ash-inclusive basis.

The pH value was determined by mixing 50 g of alfalfa materials with 200 mL of distilled water and storing this at 4°C for 18 h. The mixture was filled, and the pH value of the filtrate was measured at 15°C using a glass electrode pH meter (HI221, Hanna Ltd., Rome, Italy). The FC of alfalfa material was predicted according to the method reported by Addah et al. (2011), as follows: FC = DM% + 8 × WSC g/kg DM÷BC mEq/kg DM. FC indicates whether fresh forage is easy or difficult to be ensiled (FC > 45 = easy, FC <35 = difficult to ensile). Freeze-dried and ground alfalfa samples were used to determine the contents of monosaccharides (glucose, fructose, arabinose, and xylose) and disaccharides. The sugars in fresh alfalfa were extracted with water and measured by Agilent HPLC 1260 equipped with a chromatographic column (SP0810 sugar, Shodex, Inc. Japan) and refractive index detector. HPLC grade water was used as the mobile phase at 80°C and the flow rate was 0.75 mL/min. The AOAC-984.13 (2000) method was used to analyze the crude protein of alfalfa materials.

After 140 days of ensiling, the silo was opened, and the top layer (5 cm) of silage was removed. The silage from a depth of 5 to 10 cm at the center was then loaded out and placed in a clean box to be thoroughly mixed. A sample weighing 200 grams was selected to measure its nutrient value. The DM, crude protein, NDF, ADF, ADL, WSC, sugars, and microbes in the ensiled alfalfa were measured with the same methods as those in the fresh alfalfa materials. According to the formula reported by Porter et al. (1995), DM loss was calculated using the corrected DM content. The pH value, ammonia-N, alcohol, LA, and volatile fatty acids in ensiled alfalfa were determined according to the method reported by Dong et al. (2019).

The oligosaccharide in WSC was analyzed by thin-layer chromatography (TLC) according to the method of Kanaya et al. (1978) and with small changes: using a silica gel plate (Size: 100 × 100 mm; G model; Thickness of coating: 0.20–0.25 mm) as chromatophore; using N-butanol: acetic acid: water (2:1:1, V/V) as a solvent system; and using ethanol: sulfuric acid (4:1, V/V) for detection.

### 2.3 Statistical analyses

This study utilized IBM SPSS Statistics software (specifically, the IBM SPSS 22.0 version for Windows) for statistical analysis. Using one-way analysis of variance (ANOVA, General Linear Models), the effects of treatments on the degradation of alfalfa lignocellulose, sugar profile, fermentation characteristics, and CWL of alfalfa were evaluated. The means were then compared for significance using Tukey’s test at *p* < 0.05. Trends were discussed at 0.05 ≤ *p* ≤ 0.10.

## 3 Results and discussion

### 3.1 Combined cellulase gene-engineered MG1363 increased lignocellulose degradation

After ensiling for 140 d, treatments influenced most lignocellulosic compositions of alfalfa (*p* < 0.05), except for ADL (*p* > 0.05) (Table 1). This was because the ADL in lignocellulose was the most difficult to degrade during ensiling relative to other lignocellulosic components (Guo et al., 2014); subsequently, no change in ADL content occurred. Similar results were reported by Liu et al. (2019) and Li et al. (2018a): EN and LPEN treatments had lower NDF (*p* < 0.001 and *p* = 0.011), ADF (*p* < 0.001 and *p* = 0.028), cellulose (*p* < 0.001 and *p* = 0.003), hemicellulose (*p* < 0.001 and *p* = 0.020), and lignocellulose (*p* < 0.001 and *p* = 0.003) than the control. This was attributed to the efficient ability of cellulase to degrade lignocellulose. The LPEN had higher NDF (*p* = 0.026), ADF (*p* = 0.020), cellulose (*p* = 0.019), hemicellulose (*p* = 0.152), and lignocellulose (*p* = 0.007) than the EN, which indicated that the synergistic effect in LPEN on lignocellulose degradation did not appear. This result was consistent with the findings reported by Kozelov et al. (2008) but disagreed with the results reported by Li et al. (2018). One reason for the difference in various studies was the DM content and pH value in ensiled alfalfa. Generally, ensiled forage with high-DM content caused higher pH than that with low-DM content, and some cellulase had a great ability to degrade lignocellulose at a high pH (McDonald, 1981; Lan et al., 2013). Therefore, in practical alfalfa production, adjusting the DM content and pH value of ensiled alfalfa can enhance the activity of cellulase, thereby promoting the degradation of lignocellulose and improving the digestibility and nutritional value of alfalfa. Considering the higher (*p* < 0.05) pH in EN than in LPEN (Table 2), the pH value is a key limiting factor for the LPEN in the process of converting lignocellulose into sugars. Similar to the LPEN, HT2 had weak lignocellulose degradation relative to the EN, evidenced by higher NDF (*p* = 0.016), ADF (*p* = 0.006), cellulose (*p* < 0.001), hemicellulose (*p* = 0.225), and lignocellulose (*p* = 0.004). This was because the amount and activity of cellulase secreted by HT2 were insufficient and weak, similar to that in LPEN. Colombatto et al. (2004) and Han et al. (2023) reported that lignocellulose degradation depended on the cellulase level and acidity during ensiling.

**Table 1:** Chemical compositions of alfalfa before and after ensiling for 140 d^a^.

Items^b^	DM (g/kg FW)	CP	NDF	ADF	ADL	Cellulose	Hemicellulose	Lignocellulose
		(g/kg DM)						
FAM	279	229	431	308	110	198	123	431
CON	236^e^	237	390^a^	277^a^	100	177^a^	113^a^^b^	390^a^
EN	247^de^	227	335^c^	242^d^	97.9	144^d^	93.2^d^	335^d^
LPEN	254^b^^cd^	246	361^b^	260^b^^c^	102	158^b^^c^	101^cd^	361^b^
HT2	251^cd^	233	363^b^	264^a^^b^^c^	94.8	169^a^^b^	99.4^cd^	363^b^
HT3	271^a^	237	364^b^	251^cd^	90.8	161^b^^c^	114^a^^b^	365^b^
HT4	249^cd^	245	360^b^	252^cd^	98.5	154^cd^	108^b^^c^	360^b^
HT5	250^cd^	244	340^b^^c^	250^cd^	90.8	159^b^^c^	90.6^d^	340^cd^
C1B1	265^a^^b^	242	357^b^^c^	257^b^^cd^	109	148^cd^	100^cd^	357^b^^c^
E1B1	255^b^^cd^	235	395^a^	272^a^^b^	97.7	174^a^	123^a^	394^a^
E1C1	261^a^^b^^c^	239	349^b^^c^	257^b^^cd^	85.8	171^a^^b^	92.9^d^	349^b^^cd^
SEM	2.5	4.9	7.7	5.2	5.5	4.1	3.5	6.3
*p*-value	<0.001	0.179	<0.001	0.002	0.230	<0.001	<0.001	<0.001

a Before ensiling means that the alfalfa material is sampled after harvesting from the field for 2 h and is not fermented in anaerobic silos. After ensiling means that the alfalfa material is fermented in an anaerobic silo for 140 d and sampled after the silo is opened.

b Means in a column without a common superscript letter differed (*p* < 0.05) as analyzed by one-way ANOVA and the Tukey test.

CP, crude protein; DM, dry matter; NDF, neutral detergent fiber assayed with a heat-stable amylase and expressed inclusive of residual ash; ADF, acid detergent fiber expressed inclusive of residual ash; ADL, acid detergent lignin; FAM, fresh alfalfa material; Control, alfalfa ensiled without treatments; EN, cellulase; LPEN, the combination of *Lactobacillus plantarum* and cellulase; Lignocellulose, the sum of ADL, cellulose, and hemicellulose; HT2, HT3, HT4, HT5, E1C1, E1B1, and C1B1, combined engineered *Lactococcus lactis* subsp. lactis MG1363 strains containing *egl*3, *cbh*2, and *bgl*1 gene mixed at proportions of 1:1:1, 2:1:1, 1:2:1, 1:1:2, 1:1:0, 1:0:1, and 0:1:1, separately; SEM, standard error of the means.

Different little superscript letters “a–e” in the same column indicated significant differences among treatments at *p* < 0.05.

**Table 2:** Fermentative characteristics and microbial compositions of alfalfa before and after ensiling for 140 d^a^.

Items^b^	pH	Lactic acid	Acetic acid	Propionic acid	Butyric acid	Alcohol	LA/AA	NH_3_-N (g/kg N)	DM loss (%)	Aerobic bacteria	LAB	Clostridia spore
		(g/kg DM)								(lg cfu/g FM)		
FAM	6.01	/	/	/	/	/	/	/	/	6.97	3.40	4.30
Control	5.33^a^	42.44^f^	15.7^a^	0.58^b^	1.11	6.97^a^	2.70^d^	150^a^	2.14^a^	6.05^a^	8.69^a^^b^	5.15^a^^b^
EN	4.86^b^	58.26^cd^	15.7^a^	0.00^c^	1.18	3.30^b^	3.73^d^	135^b^	1.82^b^	5.74^a^^b^	8.24^a^^b^^c^	5.33^a^
LPEN	4.61^c^	61.54^c^	8.21^b^^c^	0.00^c^	1.31	3.00^b^	7.50^a^^b^	49.3^e^	1.56^c^	5.38^cd^	7.80^b^^cd^	5.16^a^^b^
HT2	4.70^b^^c^	72.25^b^	10.46^b^	0.00^c^	1.73	3.74^b^	6.91^a^^b^^c^	75.5^c^	1.67^c^	5.18^c^	8.90^a^	4.64^c^
HT3	4.69c	57.16^cd^	8.19^b^^c^	0.42^b^	1.24	2.77^b^	7.01^a^^b^^c^	58.7^de^	1.56^c^	5.56^b^^c^	7.20^d^	4.96^b^
HT4	4.71^b^^c^	79.85^a^	10.05^b^^c^	0.91^a^	1.64	3.41^b^	7.98^a^	75.4^c^	1.56^c^	5.64^a^^b^^c^	7.84^b^^cd^	5.25^a^^b^
HT5	4.71^b^^c^	57.98^cd^	9.11^b^^c^	0.38^b^	1.60	3.08^b^	6.37^b^^c^	64.6^cd^	1.63^c^	5.62^a^^b^^c^	7.46^cd^	5.08^a^^b^
E1C1	4.72^b^^c^	53.85^de^	8.59^b^^c^	0.00^c^	1.36	2.63^b^	6.21^c^	69.3^cd^	1.65^c^	5.53^b^^c^	7.70^cd^	5.31^a^
E1B1	4.74^b^^c^	49.89^e^	7.71^c^	0.10c	1.35	2.67^b^	6.27^c^	66.7^cd^	1.61^c^	5.61^a^^b^^c^	8.32^a^^b^^c^	5.27^a^
C1B1	4.72^b^^c^	52.45^de^	8.33^b^^c^	0.00^c^	1.37	2.67^b^	6.30^c^	67.8^cd^	1.62^c^	5.75^a^^b^	7.62^cd^	5.32^a^
SEM	0.035	2.032	0.48	0.072	0.05	0.414	0.239	2.95	0.027	0.098	0.193	0.063
*p*-value	<0.001	<0.001	<0.001	<0.001	0.164	<0.001	<0.001	<0.001	<0.001	<0.001	<0.001	<0.001

a Before ensiling means that the alfalfa material is sampled after harvesting from the field for 2 h and is not fermented in anaerobic silos. After ensiling means that the alfalfa material is fermented in an anaerobic silo for 140 d and sampled after the silo is opened.

b Means in a column without a common superscript letter differ (*p* < 0.05) as analyzed by one-way ANOVA and the Tukey test.

Cfu, colony-forming units; DM, dry matter; LA/AA, the ratio of lactic acid to acetic acid; NH_3_-N, ammonia-N; N, nitrogen; LAB, lactic acid bacteria; FAM, fresh alfalfa material; Control, alfalfa ensiled without treatments; EN, cellulase; LPEN, the combination of *Lactobacillus plantarum* and cellulase; HT2, HT3, HT4, HT5, E1C1, E1B1, and C1B1, combined engineered *Lactococcus lactis* subsp. lactis MG1363 strains containing *egl*3, *cbh*2, and *bgl*1 gene mixed at proportions of 1:1:1, 2:1:1, 1:2:1, 1:1:2, 1:1:0, 1:0:1, and 0:1:1, separately; SEM, standard error of the means; /, no detection.

Different little superscript letters “a–f” in the same column indicated significant differences among treatments at *p* < 0.05.

Compared with the control, HT3, HT4, and HT5 had lower NDF (*p* = 0.022, *p* = 0.011, and *p* < 0.001), ADF (*p* = 0.002, *p* = 0.002, and *p* = 0.001), cellulose (*p* = 0.010, *p* < 0.001, and *p* = 0.004), and lignocellulose (*p* = 0.010, *p* = 0.002 and *p* < 0.001). These outcomes indicated that these combinations could promote lignocellulose degradation. Interestingly, the degradation levels of lignocellulose in HT2 differed from those in HT3, HT4, and HT5 due to the increased expression of a cellulase component gene engineered in MG1363 relative to HT2, which altered the degradation of the lignocellulosic component. Compared with HT2, HT3 and HT4 had higher (*p* = 0.008 and *p* = 0.083) residual hemicellulose content and lower cellulose content (*p* = 0.182 and *p* = 0.014). These outcomes indicated that the increases of engineered MG1363 containing *egl*3 or *cbh*2 gene enhanced the cellulose degradation to promote the release of hemicellulose from the interweaving of hemicellulose and ADL. The result in HT3 agreed with the findings reported by Chylenski et al. (2017) due to the optimized hydrolysis in acidic pretreated lignocellulose biomass needing a high proportion of EG relative to CBH in the cellulase. The outcome in HT4 was consistent with what is observed in nature. CBH had the greatest proportion (52–80%) of the total hydrolytic enzymes secreted from *Trichoderma reesei* (Rosgaard et al., 2007). Furthermore, the increase of engineered MG1363 containing the *cbh*2 gene played a significant role in decomposing cellulose compared to that containing the *egl*3 gene, supported by a greater cellulose reduction level (*p* = 0.047) in HT3 compared to HT4. Kim et al. (2015) reported that, for acidic hydrolysis of rice straw, the role of CBH was found to be more significant than that of EG. In contrast, HT5 had lower contents of hemicellulose (*p* = 0.090), and lignocellulose (*p* = 0.017) than HT2. This was because increased BG tended to promote the degradation of hemicellulose. Indeed, some BG can degrade hemicellulose composed of xylans (Chaudhary and Tauro, 1982; Zhou et al., 2012).

Compared with the HT2, C1B1 had lower (*p* = 0.001) cellulose content. This indicated that the lack of EG did not influence cellulose degradation, which contradicted the traditional view that EG, CBH, and BG synergistically and indispensably degrade cellulose. The reason was that native bacteria on alfalfa secrete EG to help the engineered MG1363, containing *cbh*2 and *bgl*1 gene, degrade cellulose. Zhang et al. (2019) found that two EGs produced by bacteria derived from ensiled grass, such as *Paenibacillus panacisoli* SDMCC050309, could degrade carboxymethyl cellulose into cellooligosaccharides, in which cellobiose and cellotriose could be used as substrates for the growth of homofermentative LAB. In practical alfalfa production, this suggested that manipulating native bacteria could enhance the cellulose degradation during ensiling, ultimately leading to better animal performance and more efficient use of feed resources. As for the ineffective EG role of native bacteria in HT2 treatment, the reason was that the LAB in HT2 had stronger competitiveness compared to that in C1B1, supported by more LAB (*p* < 0.001) and less aerobic bacteria (*p* = 0.017) and *Clostridia* spore (*p* < 0.001) in HT2 than C1B1 (Table 2). Compared with HT2, the engineered MG1363 lacking the *cbh2* gene exhibited a significant impact on lignocellulose degradation, primarily affecting hemicellulose. This was demonstrated by higher contents of hemicellulose (*p* < 0.001) and lignocellulose (*p* = 0.001) in the E1B1 treatment. It indicated that the lack of CBH went against the degradation of hemicellulose. This is because CBH is the main component of cellulase and can degrade hemicellulose (Wei et al., 2017; Borisova et al., 2024). The lack of engineered MG1363 containing the *bgl*1 gene in E1C1 did not influence the lignocellulosic degradation relative to HT2 treatment, indicated by insignificant differences in NDF, ADF, cellulose, and hemicellulose between E1C1 and HT2 (*p* > 0.05). The reason might be that the MG1363 host could utilize the products decomposed by EG and CBH. Therefore, CBH played a more key role in lignocellulose degradation relative to EG and BG during ensiling.

### 3.2 Combined cellulase gene-engineered MG1363 altered oligosaccharide

**Figure 1:**
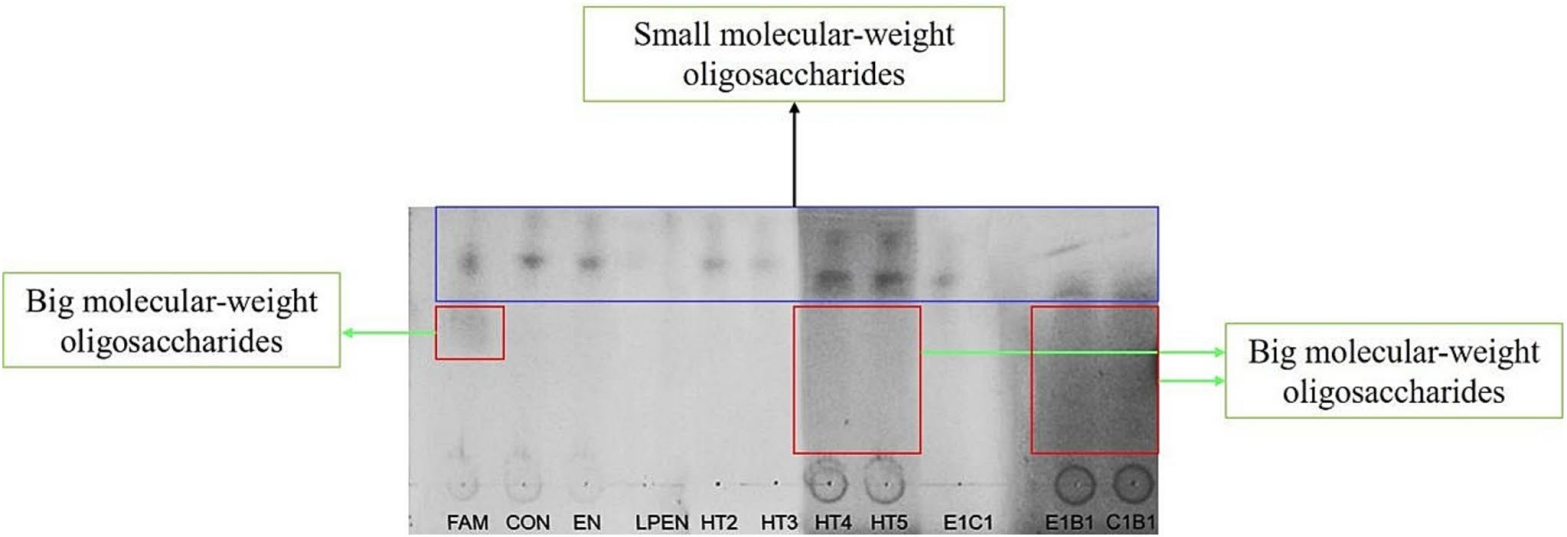
Thin-layer chromatography testing of oligosaccharide after alfalfa ensiled with treatments for 140 d. FAM, fresh alfalfa material; Control, alfalfa ensiled without treatments; EN, cellulase; LPEN, the combination of *Lactobacillus plantarum* and cellulase; HT2, HT3, HT4, HT5, E1C1, E1B1, and C1B1, combined engineered *Lactococcus lactis* subsp. lactis MG1363 strains containing *egl*3, *cbh*2, and *bgl*1 gene mixed at proportions of 1:1:1, 2:1:1, 1:2:1, 1:1:2, 1:1:0, 1:0:1, and 0:1:1, separately.

As shown in TLC, big molecular-weight oligosaccharides were found in the FAM, HT4, HT5, E1B1, and C1B1, due to shaded areas in the lanes, and there were markable small molecular-weight oligosaccharides in all samples (Figure 1). The former indicated that the broken degree of lignocellulose was greater in HT4, HT5, E1B1, and C1B1 compared with other treatments, while the latter suggested that these small molecular-weight oligosaccharides were not utilized by the MG1363 host and native LAB on alfalfa during ensiling. Fewer small molecular-weight oligosaccharides were in the LPEN compared with the EN, HT3, HT4, HT5, and the control due to its light blots. This could be explained by *Lactobacillus plantarum* having a wider fermentation profile of sugar relative to the MG1363 host and native LAB on alfalfa. Liu et al. (2020) reported that *Lactobacillus plantarum* could ferment xylose while MG1363 and *E. faecalis* could not. Interestingly, one blot of small molecular-weight oligosaccharides in E1C1 and C1B1 relative to the two blots of small molecular-weight oligosaccharides in the EN, HT2, HT3, HT4, HT5, E1B1, and the control indicated low sugar diversity in E1C1 and C1B1 due to the consumption of native bacteria or not being cut from lignocellulose. In particular, two blots of small molecular-weight oligosaccharides in the HT4 and HT5 were darker relative to those in the EN, LPEN, HT2, HT3, and the control. This indicated that more small-molecular oligosaccharides were released from lignocellulose but could not be fermented in treatments of HT4 and HT5.

### 3.3 Various sugar profiles caused by combined cellulase gene-engineered MG1363

As shown in Table 3, the control had lower WSC than EN (*p* = 0.001), LPEN (*p* = 0.001), HT2 (*p* = 0.067), and HT3 (*p* < 0.001) after ensiling for 140 d, which inferred that more WSC was released from lignocellulose due to lower lignocellulose in EN (*p* < 0.001), LPEN (*p* = 0.005), and HT2 (*p* = 0.010) (Table 1). In contrast, there was lower WSC content in HT5 (*p* < 0.001) compared with the control, and HT4, C1B1, E1B1, and E1C1 had approximately similar WSC to the control (*p* > 0.05). The former resulted from more sugar being fermented by the MG1363 host to increase the LA content. This was supported by fewer NDF (*p* < 0.001), ADF (*p* = 0.001), cellulose (*p* = 0.004), and hemicellulose (*p* < 0.001) and more LA (*p* < 0.001) in HT5 than the control (Table 2). Interestingly, the reasons for the low WSC in HT4, C1B1, E1B1, E1C1, and the control were different. Similar to HT5, low WSC content in HT4 was because the WSC released from lignocellulose degradation could be fermented by the MG1363 host during ensiling due to the extra-engineered MG1363 containing the *cbh*2 gene. Furthermore, the WSC consumption ability of HT5 was stronger than HT4. A detailed reason for this is unknown since there was no difference in the bacteria compositions, and thus it needs further study in the future. Conversely, the low WSC in the control and E1B1 indicated that little fermentable WSC was derived from the lignocellulose degradation due to the high lignocellulose composition (Table 1). Consequently, the inherent WSC in alfalfa was consumed in the control and E1B1. The low WSC in the C1B1 and E1C1 was due to the fact that the WSC that originated from lignocellulose degradation could be fermented, supported by the lower lignocellulose (*p* < 0.001) and higher LA content (*p* = 0.002 and *p* < 0.001) than the control (Table 2).

**Table 3:** Sugars of alfalfa before and after ensiling for 140 d^a^.

Items^b^	Water-soluble carbohydrates (kg DM)	Disaccharide (g/kg DM)	Monosaccharide (g/kg DM)			
			Glucose	Xylose	Arabinose	Fructose
FAM	84.3	24.0	10.7	13.7	0.21	10.5
CON	35.9^b^^c^	0.00^f^	/	16.8^c^	0.00^c^	/
EN	42.8^a^	7.59^c^	/	19.1^a^	0.24^a^	/
LPEN	42.8^a^	9.31^b^	/	17.7^b^^c^	0.24^a^	/
HT2	39.4^a^^b^	8.64^b^^c^	/	19.2^a^	0.08^b^^c^	/
HT3	43.7^a^	10.9^a^	/	19.4^a^	0.02^c^	0.08
HT4	35.3^b^^c^	1.02^ef^	/	18.5^a^^b^	0.20^a^	/
HT5	26.2^d^	8.28^b^^c^	/	18.6^a^^b^	0.04^b^^c^	0.06
C1B1	34.4^c^	4.13^d^	/	18.9^a^	0.02^c^	1.04
E1B1	36.0^b^^c^	4.52^d^	/	18.7^a^^b^	0.08^b^^c^	/
E1C1	34.9^b^^c^	2.24^e^	/	18.6^a^^b^	0.14^c^	0.10
SEM	1.42	0.544	/	0.33	0.034	/
*p*-value	<0.001	<0.001	/	<0.001	<0.001	/

a Before ensiling means that the alfalfa material is sampled after harvesting from the field for 2 h and is not fermented in anaerobic silos. After ensiling means that the alfalfa material is fermented in an anaerobic silo for 140 d and sampled after the silo is opened.

b Means in a column without a common superscript letter differed (*p* < 0.05) as analyzed by one-way ANOVA and the Tukey test.

FAM, fresh alfalfa material; Control, alfalfa ensiled without treatments; EN, cellulase; LPEN, the combination of *Lactobacillus plantarum* and cellulase; HT2, HT3, HT4, HT5, E1C1, E1B1, and C1B1, combined engineered *Lactococcus lactis* subsp. lactis MG1363 strains containing *egl*3, *cbh*2, and *bgl*1 gene mixed at proportions of 1:1:1, 2:1:1, 1:2:1, 1:1:2, 1:1:0, 1:0:1, and 0:1:1, separately; SEM, standard error of the means; /, below the detection limitation or without statistical analyses.

Compared with the control, most of the treatments had higher disaccharides (*p* < 0.05), except for HT4 (*p* > 0.05). The explanation for the former was that disaccharide was formed from lignocellulose degradation and was not consumed by *L. latics* and some native bacteria on the alfalfa. The latter indicated that the HT4 not only strongly converted lignocellulose into disaccharide but also had the strongest ability to consume the disaccharide. The extra CBH secreted by the engineered MG1363 strain containing the *cbh*2 gene in HT4 could attack the non-reducing end of the crystalline cellulose created by EG and release cellobiose (Chylenski et al., 2017), which could be further cut by BG into monosaccharide and was fermented by the *L. latics* host to produce more LA than other treatments (*p* < 0.001) (Table 2). The lack of an engineered MG1363 strain containing the *cbh*2 gene in E1B1 resulted in higher disaccharide content than HT4 (*p* < 0.001). It was hard to explain the disaccharide source. A proposed reason might be that the end of the crystalline cellulose created by EG could be digested into disaccharides in an acidic ensiling environment. The higher (*p* < 0.001) disaccharide content in EN, HT2, HT3, HT5, and LPEN than in HT4 might be because the consumption level of disaccharide in the above treatments was weaker relative to HT4. On the other hand, HT3 had the highest disaccharide content than other treatments (*p* < 0.001). This might result from the extra EG secreted by the engineered MG1363 strain containing the *egl*3 gene in HT3, which could produce quite a number of the ends of the cellulose crystallinity to restrain the CBH and BG activity. Some studies reported that cellulase adsorption generally declined as cellulose crystallinity increased (Yang et al., 2011). Crystallinity could greatly impact the adsorption of Cel7A (CBHI), leading to a decrease in hydrolysis (Jeoh et al., 2007). Cello-oligosaccharide substrates inhibited the activity of BG (Kawai et al., 2004; Bohlin et al., 2013; Teugjas and Väljamäe, 2013). Consequently, the channel of cellobiose converting into glucose was blocked by disaccharides, which accumulated in HT3. Interestingly, LPEN had higher disaccharide contents than EN (*p* < 0.05). This indicated that the ability of the native LAB on the alfalfa material to ferment disaccharides was weaker than that of *Lactobacillus plantarum*.

**Figure 2:**
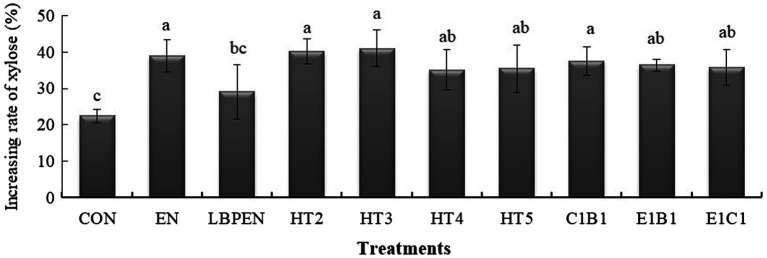
The effects of treatments on the increasing rate of xylose after ensiling for 140 d. The increasing rate of xylose was calculated by the formula: Increasing rate of xylose = (Xylose _ensiled alfalfa_–Xylose _raw alfalfa material_) ÷ Xylose _raw alfalfa material_ ×100%. Bars with different letters (a-c) indicated the difference among the treatments at *p* < 0.05 after being analyzed by one-way ANOVA and the Tukey test. CON, alfalfa ensiled without treatments; EN, cellulase; LPEN, the combination of *Lactobacillus plantarum* and cellulase; HT2, HT3, HT4, HT5, E1C1, E1B1, and C1B1, combined engineered *Lactococcus lactis* subsp. lactis MG1363 strains containing *egl*3, *cbh*2, and *bgl*1 gene mixed at proportions of 1:1:1, 2:1:1, 1:2:1, 1:1:2, 1:1:0, 1:0:1, and 0:1:1, separately.

All treatments had higher xylose than the control (*p* < 0.05), except for LPEN (Figure 2). The reasons for the former were as follows: ① Xylose was hardly metabolized by the MG1363 (Erlandson et al., 2000); ② Some native LAB in the control could utilize xylose. Cai (1999) found that some isolated native LAB from forage crops, such as *Enterococcus casseliflavus, Enterococcus gallinarum*, and *Enterococcus mundtii*, could ferment xylose; and ③ EN degraded more hemicellulose (*p* < 0.001) into xylose than the control. The reason for this was that *Lactobacillus plantarum* could ferment xylose. Our previous study reported that the MG1363 had a weaker ability to ferment xylose relative to *Lactobacillus plantarum* (Liu et al., 2019). Compared with the control, higher arabinose content in HT4 (*p* < 0.001), EN (*p* < 0.001), and LPEN (*p* < 0.001) indicated that hemicellulose was degraded into arabinose due to lower hemicellulose content (*p* = 0.384, *p* < 0.001, and *p* < 0.001) than the control and the extra engineered MG1363 strain containing *cbh*2 gene in HT4 relative to HT2, HT3, HT5, C1B1, E1B1, and E1C1 could cut hemicellulose to release arabinose.

### 3.4 Combined cellulase gene-engineered MG1363 changed the fermentative characteristics and microbial compositions of ensiled alfalfa

As shown in Table 2, compared with the control, all treatments improved the fermentation due to the lower pH (*p* < 0.05), low LA content (0.1 < *p* < 0.05) and ammonia-N (*p* < 0.05), and DM loss (*p* < 0.05). It was because cellulase derived from the EN and secreted by engineered *L. latics* combination could degrade lignocellulose into the sugars to enhance the LA fermentation of *L. latics* and native LAB. Interestingly, EN promoted the LA fermentation but did not decrease the AA content (*p* = 0.984) compared with the control. This was because that EN promoted the hetero-LA fermentation of native LAB on alfalfa. In this study, hetero-LA fermentation was associated with high DM loss and concentrations of acetic acid and ammonia-N during ensiling relative to homolactic fermentation, supported by previous studies (Driehuis et al., 1999; Blajman et al., 2020). Compared with EN, DM loss (*p* < 0.05) and AA content (*p* < 0.05) were decreased by the supplementations of HT2, HT3, HT4, HT5, LPEN, E1C1, E1B1, and C1B1, accompanied by higher LA/AA (*p* < 0.05) and lower ammonia-N (*p* < 0.05). This result indicated that the homolactic fermentation was more vigorous in those treatments relative to in EN. Liu et al. (2019) demonstrated that engineered homo-fermentative LAB strains containing cellulase genes or the combination of wild-type MG1363 and cellulase had stronger homolactic fermentation in ensiled alfalfa compared with adding cellulase alone. Tian et al. (2014) and Xing et al. (2009) found that *Lactobacillus plantarum* combined with cellulase promoted homolactic fermentation and inhibited the degradation of protein to produce ammonia-N.

**Figure 3:**
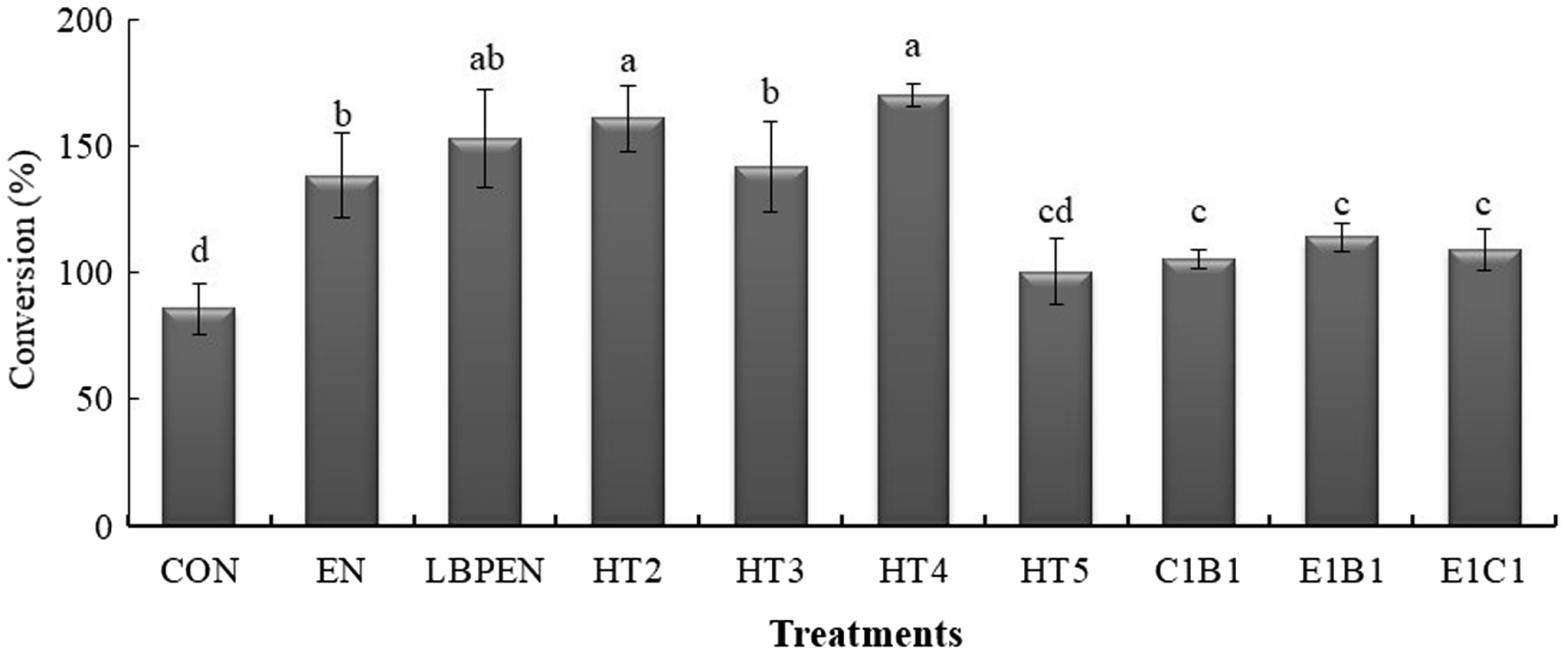
The conversions of water-soluble carbohydrates to lactic acid in ensiled alfalfa. Means in a bar without a common letter differ (*p* < 0.05) from each treatment after being analyzed by one-way ANOVA and the Tukey test. CON, alfalfa ensiled without treatments; EN, cellulase; LPEN, the combination of *Lactobacillus plantarum* and cellulase; HT2, HT3, HT4, HT5, E1C1, E1B1, and C1B1, combined engineered *Lactococcus lactis* subsp. lactis MG1363 strains containing *egl*3, *cbh*2, and *bgl*1 gene mixed at proportions of 1:1:1, 2:1:1, 1:2:1, 1:1:2, 1:1:0, 1:0:1, and 0:1:1, separately.

Interestingly, compared with the HT2, increasing engineered MG1363 containing one gene of cellulase component altered the LA fermentation. The HT4 had a higher LA content than HT2 (*p* = 0.013), indicating that increasing engineered MG1363 with the *cbh*2 gene had a better effect on promoting LA fermentation. In addition, relative to HT2, increasing engineered MG1363 containing *cbh*2 gene was better than increasing engineered MG1363 with *egl*3 or *bgl*1 gene on increasing LA content (*p* < 0.001), supported by HT4 having higher LA content (*p* < 0.05) and CWL (*p* = 0.004 and *p* < 0.001) than HT3 and HT5 (Figure 3). This was attributed to the CBH being the key cellulase component for degrading lignocellulose into fermentable sugar, followed by MG1363 host-converted sugar into more LA, demonstrated by lower cellulose (*p* = 0.014), fewer fermentable disaccharides (*p* < 0.001), and higher LA content (*p* = 0.013) in HT4 than HT2. Therefore, CBH cellobiohydrolase better cooperated with MG1363 to ferment lignocellulose into LA relative to endoglucanase and *β*-glucosidase.

Although there was lower LA content (*p* < 0.001) and CWL (*p* = 0.038 and *p* < 0.001) in HT3 and HT5 relative to HT2, the mechanisms of altering LA fermentation were different in HT3 and HT5. Compared with HT2, the HT3 containing engineered MG1363 with *egl*3 gene restrained the channel of fermenting sugar into LA rather than converting lignocellulose into sugar. It was because of its approximate residual WSC (*p* > 0.05) and lignocellulose content (*p* = 0.788) and lower LA content (*p* < 0.001) and CWL relative to HT2. In contrast, HT5 containing engineered MG1363 with *bgl*1 gene did not restrain the channel of converting lignocellulose into sugar due to its lower lignocellulose (*p* = 0.017) relative to HT2 but disturbed the channel of fermenting sugar into LA, supported by its lower contents of residual WSC (*p* < 0.001), LA (*p* < 0.001), and CWL (*p* < 0.001) and more propionic acid relative to HT2 (*p* = 0.001). On the other hand, LPEN had lower LA content (*p* = 0.001), approximate CWL (*p* = 0.374), residual WSC (*p* = 0.072), and lignocellulose (*p* = 0.795) relative to HT2, and had weaker (*p* = 0.004) lignocellulose degradation relative to EN due to the lower pH (*p* < 0.05), which indicated that the pH in the channel of fermenting sugars into LA was the key limiting factor for lignocellulose saccharification in LPEN. This might be one explanation to clarify the weak or no effect on reducing the contents of NDF and ADF and improving the LA fermentation in ensiled alfalfa treated with the combination of cellulase and LAB in previous studies (Kozelov et al., 2008; Lynch et al., 2014). Therefore, the pH adaptability of cellulase for lignocellulose saccharification and ensuring synergy with LAB strains during fermenting lignocellulosic forage to produce LA are key considerations.

Accordingly, the lack of any engineered MG1363 containing one gene of cellulase component in C1B1, E1B1, or E1C1 had lower LA production (*p* < 0.001) compared with HT2. This outcome could be explained by the following reasons: ① fewer LAB numbers in C1B1 (*p* < 0.001), E1B1 (*p* = 0.043), and E1C1 (*p* < 0.001) than in HT2; ② the insufficient unblocked channel of converting lignocellulose into sugar was in E1B1 due to its higher residual lignocellulose (*p* = 0.001) than in HT2; and ③ the insufficient unblocked channels of converting sugar into LA were in C1B1 and E1C1 due to their lower LA content (*p* < 0.001) and CWL (*p* < 0.001), which was not the case in HT2. Therefore, this study optimized the combination of engineered MG1363 containing cellulase component genes to promote LA fermentation, clarified the key cellulase component playing a synergistic role with LAB in fermenting alfalfa lignocellulose into LA, and identified the synergetic disadvantage of cellulase combined with LAB for LA fermentation in ensiled alfalfa feedstock.

## 4 Conclusion

HT4 represents the optimal combination proportions of engineered MG1363 containing the *bgl1*, *cbh2*, and *egl3* genes at a ratio of 1:2:1, which could enhance LA fermentation. The combination of cellulase gene-engineered MG1363 clarified that the CBH was more crucial in converting lignocellulose into fermentable sugar than EG and BG and cooperated with the MG1363 host to produce more LA content. The pH in the channel of fermenting sugars into LA was the key limiting factor for lignocellulose saccharification in the combination of *Lactobacillus plantarum* and cellulase in ensiled alfalfa. This study could benefit the development of LA production in fermenting lignocellulosic biomass via optimizing the combination of cellulase gene-engineered LAB.

## Data Availability

The raw data supporting the conclusions of this article will be made available by the authors, without undue reservation.

## Footnotes

^1^ https://www.reportsanddata.com/report-detail/lactic-acid-market
